# Ileo-Ileal Intussusception Caused by a Meckel’s Diverticulum With Ectopic Pancreatic Tissue: A Case Report

**DOI:** 10.7759/cureus.51888

**Published:** 2024-01-08

**Authors:** Julio A Palma Zapata, Alondra E Llamas Domínguez, Zhenia L Fernández Álvarez, Juliana Palma Zapata

**Affiliations:** 1 Medical Didactic Unit, Autonomous University of Aguascalientes, Aguascalientes, MEX; 2 Pediatric Service, Institute for Social Security and Services for State Workers, Aguascalientes, MEX; 3 Department of Medicine, Health Sciences Center, Autonomous University of Aguascalientes, Aguascalientes, MEX

**Keywords:** meckel's diverticulum, meckel's diverticulum complications, intestinal obstruction, ectopic pancreas, intususcepción

## Abstract

A Meckel's diverticulum (MD) is an embryonic remnant of the omphalomesenteric duct. Although most MDs are usually asymptomatic, pediatric patients tend to present serious complications more frequently (4-25% of cases), mainly in digestive tract bleeding, intestinal obstruction, and perforation, which have a high potential to compromise the patient's life. An ectopic pancreas (EP) is pancreatic tissue found outside the pancreas, usually in the stomach, duodenum, and jejunum. It is typically asymptomatic but can increase the risk of complications in the MD. A clinical case of a female infant with an MD complicated with bleeding and ileo-ileal intussusception is presented, in which the histopathological finding of type 1 ectopic pancreatic tissue was also found based on the Heinrich classification, being an entity uncommon in our environment. An EP arising within an MD is infrequent, requiring clinical attention and timely preoperative diagnosis to prevent and treat associated severe complications. This continues to be a superior challenge for the clinician and requires a multidisciplinary team for comprehensive treatment.

## Introduction

A Meckel's diverticulum (MD) is a true diverticulum that results as a remnant of the omphalomesenteric or vitelline duct derived from an incomplete regression of the same during weeks five to seven of gestation. It is located on the antimesenteric border of the small intestine, and its most frequent location is in the distal ileum, 40-60 cm away from the ileocecal valve [1]. It is the most common congenital gastrointestinal anomaly; it has a prevalence of approximately 2% of the world's population [2,3]. A systematic review authored by Hansen et al. has revealed that the prevalence of MDs varies between 0.3% and 2.9% in the general population, as determined by eight studies [4]; however, it is difficult to specify the exact number because most patients are asymptomatic. In Mexico, a prevalence of 1.2% is reported. More than half of the cases occur before the age of four years; however, the classic age of presentation of a symptomatic MD is 2.5 years [4,5].

Although the majority of MDs are usually asymptomatic, pediatric patients are more likely to experience severe complications (4-25% of cases). The complications that they experience are mainly related to the digestive tract, such as bleeding, intestinal obstruction, and perforation. These complications can be life-threatening for the patient [3,4,6]. In addition, the presence of ectopic tissue within the MD is a significant risk factor for complications. It is estimated that 12% of patients present ectopic tissue in MDs, and 43% of them are symptomatic [7].

The most common ectopic tissues involved in MDs are gastric in 62% and pancreatic in 6% of the cases; others found less frequently include the jejunal tissue, duodenal secretory mucosa (Brunner's glands), colonic, rectal, endometrial, and even hepatic tissues [8].

An ectopic pancreas (EP) refers to a pancreatic tissue that is not anatomically, vascularly, or neurally connected to the pancreas [8,9]. Its etiology is unknown; however, proposed theories suggest an error during embryological development in which the foregut rotation and dorsal and ventral pancreatic bud fusion separate, causing small segments to migrate and develop in abnormal locations within the digestive system. The most common sites for finding EP are the stomach, duodenum, jejunum, and rarely the ileum and MD [10]. 

An EP is also an entity that is asymptomatic, but it poses a higher risk of complications for patients with MDs. This is because the EP actively secretes pancreatic juice, which can lead to ulceration, bleeding, inflammation, and even intestinal intussusception [11].

Here, we report an unusual case of an MD complicated by bleeding and ileo-ileal intestinal invagination and discuss factors responsible for this condition, the diagnostic approach, therapeutic decisions, and the dissemination of a rare case in our current environment. It highlights the importance of considering Meckel's diverticulum as a differential diagnosis in every patient with an acute abdomen in the pediatric population to avoid serious complications.

## Case presentation

A 14-month-old female patient with no significant medical history was brought to the emergency department. The patient had been experiencing persistent vomiting of biliary content for more than 36 hours, along with a fever that peaked at 101.3ºF (38.5°C). In addition, the patient had currant jelly stool with a consistency of Bristol six (mushy stool in the form of fluffy pieces with ragged edges) on 12 occasions. 

**Figure 1:**
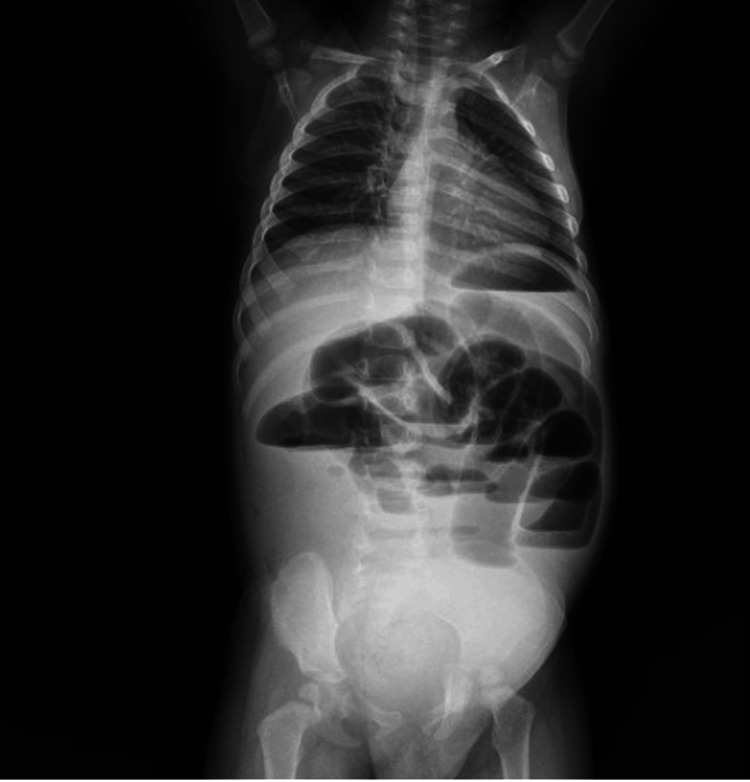
Abdominal X-ray anteroposterior projection in the standing position The abdominal X-ray reveals intestinal loop dilatation and gas-filled loops.

Upon admission, the patient's vitals were as follows: blood pressure 90/55 mmHg, heart rate 150 bpm, respiratory rate 35 rpm, temperature 101.4ºF (38.5°C), oxygen saturation 95% with a fraction of inspired oxygen (FiO_2_) 21%, and weight 19.16 pounds (8.69 kg). On clinical examination, the patient presented with moderate dehydration and exhibited consciousness and orientation. The lungs were well ventilated with no signs of respiratory distress, while the precordium had intensified heart sounds without murmurs. The patient had a distended and painful abdomen that showed signs of peritoneal irritation and fighting peristalsis, and a positive rectal examination was performed for blood with fecal matter. An abdominal X-ray was taken in a standing position, revealing signs of intestinal obstruction, such as intestinal loops dilatation and gas-filled loops (Figure 1). A nasogastric tube was placed, which drained biliary and fecaloid contents.

The admission laboratory results are presented in Table 1.

**Table 1:** Laboratory data HB, hemoglobin; Hto, hematocrit; WBC, white blood cells; Lymphs, lymphocytes; Monos, monocytes; Baso, basophils; CRP, C-reactive protein

Test	Result	Normal value range for age
HB	14.5 g/dl	10.7-13.9 g/dl
Hto	44%	33-36%
Platelet count	592,900/μL	150,000–450,000/μL
WBC	13.2 x 10^3^/µL	6.0–17.5 x 10^3^/µL
Neutrophils	50%	37-71%
Lymps	34%	17-67%
Monos	13%	0-5%
Baso	1%	0-3%
Glucose	82 mg/dL	70-110 mg/dL
Creatinine	0.4 mg/dL	0.12–1.06 mg/dL
Sodium	134 mmol/L	136–145 mmol/L
Potassium	5.1 mmol/L	3.5–5.5 mmol/L
Chloride	89 mmol/L	95–105 mmol/L
Calcium	9.6 mg/dL	8.7–9.8 mg/dL
CRP	24.5 mg/L	< 0.8 mg/L
Procalcitonin	0.29 ng/mL	<0.5 ng/mL

**Figure 2:**
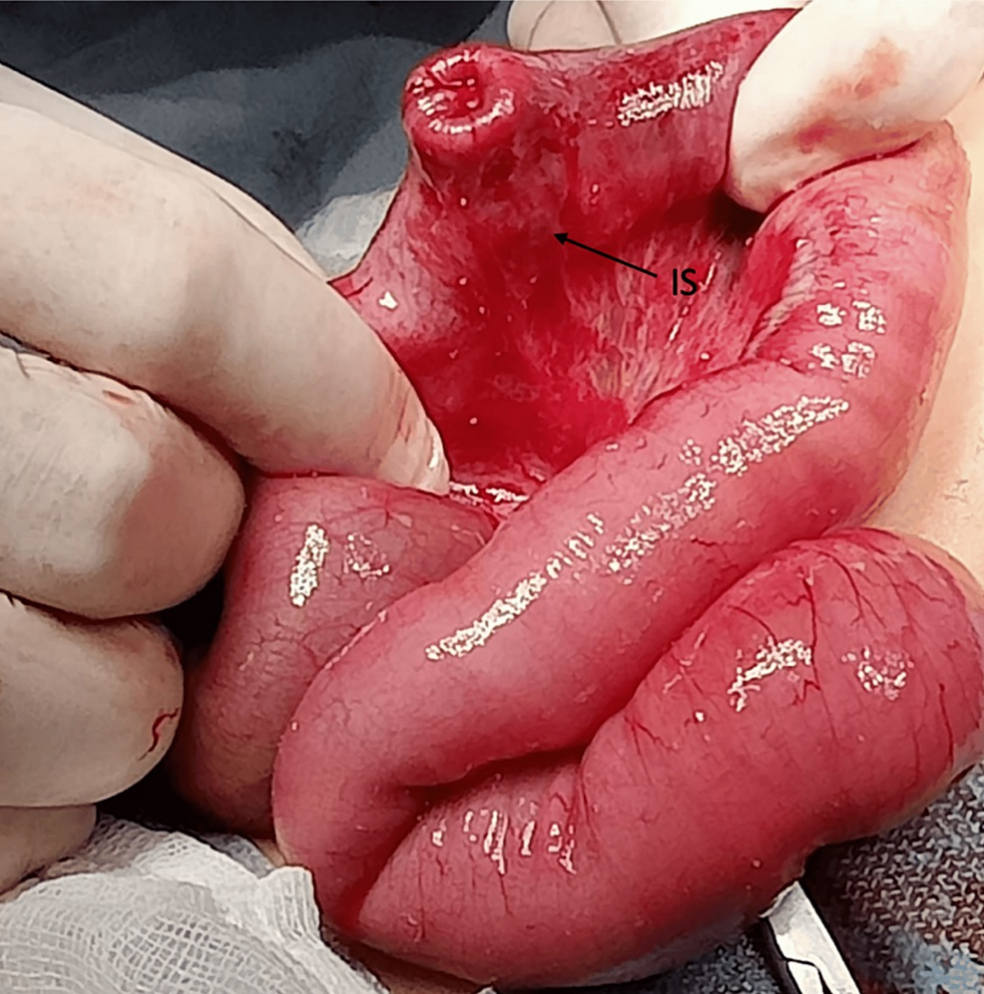
Intraoperative findings Signs of ischemia (IS) and edema are observed in the ileo-ileal intestinal invagination caused by Meckel's diverticulum.

A diagnostic investigation of intestinal intussusception was performed using abdominal ultrasound. The findings revealed a large amount of intestinal gas within the intestinal loops. Therefore, it was not possible to visualize signs of invagination. Due to the suspected intestinal intussusception and the patient's age, a decision was made to perform an exploratory laparotomy. During the procedure, an ileo-ileal intussusception was discovered approximately 30 cm from the ileocecal valve, resulting from a Meckel's diverticulum with a pedunculated polyp (Figure 2).

The procedure involved disinvagination of the intussusception by taxis, resection of the diverticulum, and end-to-end intestine anastomosis.

**Figure 3:**
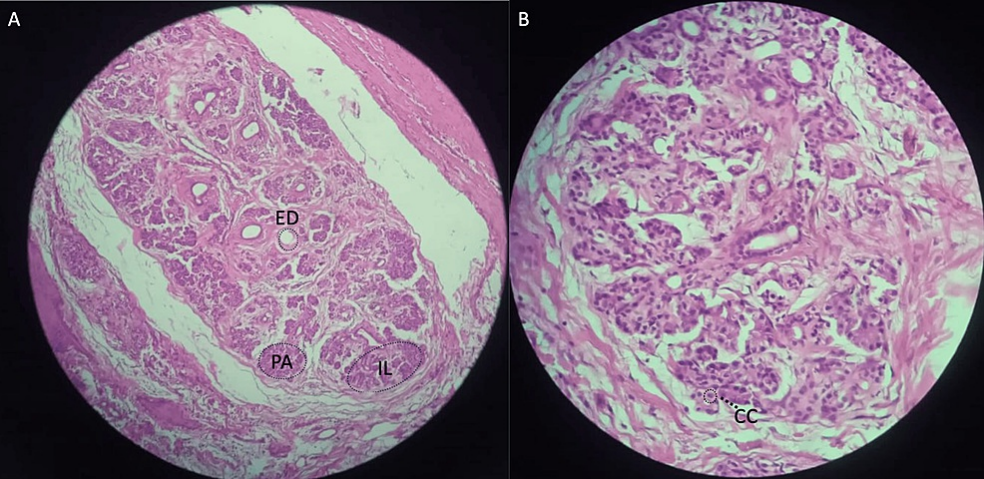
Microscopy image H&E staining of Meckel's diverticulum. A) (10x), B) (40x) It reveals the presence of glandular structures that consist of cuboidal cells, which collectively form the pancreatic acini (PA) exhibiting intense hematoxylin staining. In addition, the image reveals the existence of islets of Langerhans (IL), comprising polygonal cells with voluminous and pale cytoplasm and monomorphic nuclei with classic salt and pepper chromatin. Furthermore, the pancreatic ducts are visible, lined with low cuboidal epithelium that has uniform nuclei alternating with the pancreatic acini. The tissue in question is classified as an ectopic pancreatic tissue type 1 as per the Heinrich classification, owing to the presence of centroacinous cells (CC), excretory ducts (ED), and IL.

During the histopathological examination, an MD measuring 3.5 cm x 2 cm with a whitish-yellow invaginated area was observed macroscopically. The hematoxylin and eosin staining analysis of the histological section revealed a Meckel's diverticulum with Heinrich type 1 ectopic pancreatic tissue implantation, and no signs of malignancy were found (Figure 3).

**Figure 4:**
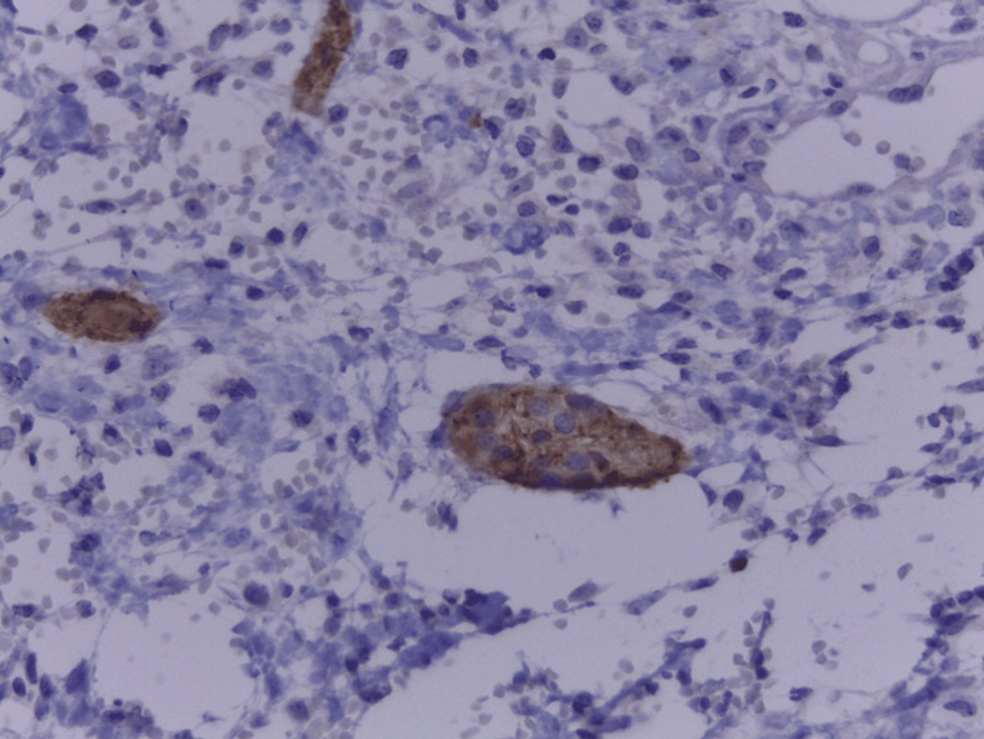
Positive synaptophysin immunostaining (10x) It exhibits a diffuse pattern of cytoplasmic decoration of the neuroendocrine cells present in the islets of Langerhans (IL). This study shows the molecular characteristics of an ectopic pancreas in Meckel's diverticulum.

The sample was also subjected to an immunohistochemical study, which confirmed that it tested positive for chromogranin and synaptophysin (Figure 4).

Following surgery, the patient was hospitalized for a period of three weeks due to the development of anastomotic dehiscence and intestinal pneumatosis. In response to the patient's condition, the medical team decided to perform an ileostomy. During the hospitalization, the patient received broad-spectrum antibiotic therapy, prolonged fasting, and parenteral nutrition. Following a period of clinical improvement, the patient was discharged. Six months later, the patient was readmitted to undergo a reversal of the ileostomy. Currently, the patient remains in good general health.

## Discussion

The MD is a remnant of the omphalomesenteric duct and is composed of all layers of the small intestine. It is more common in males, with a male/female ratio of 2:1 [3]. There have been only a few published cases of this nature in the current literature. A PubMed database search was conducted using the term "Meckel diverticulum" in combination with "ectopic tissue" and "pancreatic." Eighteen case reports in the pediatric population (children: birth-18 years) were found in English language articles published between 1979 and 2023. The MD is usually situated at a distance of 40-60 cm from the ileocecal valve. It measures approximately 5 cm in length and has a diameter of 2 cm. However, in our case, it was located 30 cm from the ileocecal valve and had a length of 3 cm and a diameter of 2 cm (Figure 2). Diverticulums that are longer than 2 cm in length are more likely to cause complications and symptomatic disease compared to smaller diverticulums. Moreover, certain risk factors, such as male gender, the presence of ectopic tissue, broad-based diverticulum, and fibrous bands attached to the diverticulum, may contribute to the development of associated symptoms [12].

The main clinical symptoms of MDs include abdominal pain, nausea, vomiting, and intestinal bleeding, which can appear as hematochezia, melena, or rectal bleeding [1]. Gastrointestinal bleeding is a crucial indicator as it could be the initial and sole indication that alerts us to suspect this condition. According to the literature, painless rectal bleeding is the typical symptom observed in children under two years of age [9].

However, the clinical presentation of this condition can vary significantly and is often secondary to the development of complications. The most common complications include gastrointestinal bleeding (30-56%), intestinal obstruction and intussusception (14-42%), diverticulitis (6-14%), perforation (7%), and neoplasia (3.2%) [1,13]. Late diagnosis of this entity can have life-threatening consequences, including intestinal gangrene, peritonitis, and sepsis [14].

The patient developed gastrointestinal bleeding caused by intestinal intussusception. The intussusception was found at an atypical location, the ileo-ileal level. It is worth noting that the most common types of intussusception are ileocolic, followed by ileo-ileocolic, ileo-ileal, and, finally, colicocolic, which are uncommon [3].

When it comes to diagnosing a complicated MD in patients, ultrasonography may only be helpful in cases where the MD involves intestinal intussusception. In such cases, an ultrasound is the recommended office study, which can reveal the "target" or "pseudokidney" sign [3]. However, some authors contend that ultrasound's ability to identify intussusception as an underlying cause of MDs is limited in less than one-third of cases. Similarly, in our situation, the abdominal ultrasound was inconclusive due to the modification of ultrasound by the presence of intussusception as a complication of MDs [11]. Contrast-enhanced computed tomography or magnetic resonance imaging can be used to detect MDs as a tubular structure in the right lower quadrant or near the umbilical region [3]. Some authors suggest that exploratory laparoscopy is the most effective diagnostic test; in most of the cases reported, invaginations resulting from MDs were diagnosed during the surgical intervention [14].

Technetium 99 scintigraphy, also known as the "Meckel scan," remains the gold standard method for the diagnosis of MDs. It has a sensitivity of 94% and a specificity of 97%. However, it may not be advantageous when diagnosing MDs with EP since TC99 has a high affinity only for the gastric mucosa [2]. In this case, due to the acute nature of the presentation and poor access to the study, it was not performed, and it was decided to opt for surgical exploration. However, an MD without ectopic gastric mucosa will not be visible on a Technetium 99 scintigraphy.

Localization of an EP within an MD is extremely rare, and ectopic tissue is typically asymptomatic unless complicated by inflammation, intestinal obstruction, intussusception, or malignant transformation (pancreatic adenocarcinoma) [15].

In the diagnosis of EP, preoperative diagnostic cabinet studies lack specificity, thus rendering histopathological study following tissue resection as the most dependable method [10].

EP are classified using the Heinrich criteria, which distinguish three types. Type 1 is characterized by the presence of acini, excretory ducts, and islets of Langerhans (IL). Type 2 presents acini and excretory ducts, while type 3 only contains excretory ducts. The most prevalent type is type 2, followed by type 1, while type 3 is the least commonly encountered [8]. In our case, the histopathological classification of the PE corresponded to Heinrich type 1, which is less frequent, with only a few reported cases (Figure 3). 

The occurrence of EP in the MD is associated with an elevated risk of complications. This is attributed to the pancreatic juice that is produced, which can result in various unfavorable outcomes, such as ulceration, bleeding, tissue inflammation, and alteration of the peristaltic rhythm. These factors also increase the likelihood of intussusception [14].

The recommended treatment for symptomatic cases of MDs is the surgical removal of the inflamed small intestine along with the rest, using either a laparoscopic or open technique. Moreover, it is advisable to explore the entire ileum through surgery to ensure the identification and removal of other ectopic segments, ulcers, and bleeding. The treatment of asymptomatic MDs is still a matter of debate and may involve either surgery or observation. However, in case it is discovered accidentally during an abdominal surgery, it is suggested to remove it [3].

## Conclusions

An MD should be considered a possible diagnosis when there is evidence of intestinal intussusception in the pediatric population with no surgical history. However, this is a rare form of secondary intussusception. The occurrence of an EP within an MD is a rare phenomenon that requires urgent clinical attention and timely preoperative diagnosis, particularly in cases of intestinal obstruction. The management of severe complications that come with these pathologies represents a significant challenge for clinicians, necessitating a multidisciplinary approach to ensure effective treatment. In cases where diagnostic tests are inconclusive, exploratory laparotomy remains the most effective diagnostic and therapeutic option, as observed in the present case.
